# ASSOCIATION OF TUMOR DEPOSITS WITH TUMOR PATHOLOGY AND SURVIVAL IN GASTRIC CANCER: A CROSS-SECTIONAL STUDY

**DOI:** 10.1590/S0004-2803.24612026-008

**Published:** 2026-09-21

**Authors:** Nasrin SARKHANI, Bahareh MEHRAMUZ, Ali Akbar SHEKARCHI, Sajad SARKHANI, Maryam JAFARZADEH, Elham SARKHANI

**Affiliations:** 1 Tabriz University of Medical Sciences, Department of Pathology, Tabriz, Iran.; 2 Tabriz University of Medical Sciences, Sina Educational, Research, and Treatment Center, Pathology at Clinical Research Development Unit, Tabriz, Iran.; 3 Azad University of Medical Sciences, School of Medicine, Tehran, Iran.; 4 Azad University of Medical Sciences, School of Medicine, Ardabil, Iran.; 5 Ardabil University of Medical Sciences, Faculty of Medicine, Department of Biochemistry, Ardabil, Iran.

**Keywords:** Stomach Neoplasms, tumor deposits, extranodal extension, neoplasm staging, prognosis, Neoplasias Gástricas, depósitos tumorais, extensão extranodal, estadiamento de neoplasias, prognóstico

## Abstract

**Background:**

Tumor deposits (TDs) are recognized as adverse prognostic factors in colorectal cancer and are incorporated as N1c in the TNM staging system. Their significance in gastric cancer (GC), however, remains less clear.

**Objective:**

This study aimed to investigate the association of TDs with pathological features and survival outcomes in GC.

**Methods:**

We conducted a cross-sectional study of patients with pathologically confirmed GC treated at two tertiary hospitals in Tabriz, Iran, between January 2020 and December 2024. Clinical and pathological data, including tumor characteristics and survival status, were collected for the participants. TDs and their characteristics were also evaluated. Associations between the presence of TDs and clinicopathological features were analyzed using logistic regression models, while overall survival was assessed using Kaplan-Meier analysis and Cox proportional hazards regression.

**Results:**

The study included 256 patients (mean age 60.7±12.3 years; 61.3% male). TDs were present in 111 patients (43.4%). In multivariable analysis, pT category (OR=7.44, 95%CI: 2.22-24.89), pN category (OR=4.63, 95%CI: 1.97-10.86), and perineural invasion (OR=5.65, 95%CI: 2.40-13.29) were significant correlates of TDs. Kaplan-Meier analysis showed 1-, 3-, and 5-year overall survival rates of 93.7%, 75.7%, and 64.9%, respectively. Patients without TDs had significantly better survival compared to those with TDs (log-rank *P*<0.001). In multivariable Cox regression analysis, histological subtype (HR=2.07, 95%CI: 1.21-3.52), pT category (HR=4.68, 95%CI: 1.37-16.00), pN category (HR=2.79, 95%CI: 1.21-6.46), and the presence of TDs (HR=2.00, 95%CI: 1.06-3.75) were identified as independent predictors of poorer overall survival.

**Conclusion:**

TDs were observed in a considerable proportion of GC cases and were associated with more aggressive pathological features and poorer survival. They may improve prognostic stratification, but further validation in larger multicenter studies is required.

## HIGHLIGHTS

• Tumor deposits (TDs) were present in 43.4% of gastric cancer patients.

• TDs were associated with advanced pT and pN categories and perineural invasion.

• The presence of TDs independently correlated with poorer overall survival.

• TDs offer additional prognostic information in gastric cancer, supporting further multicenter validation.

## RESUMO

**Contexto:**

Os depósitos tumorais (DTs) são reconhecidos como fatores prognósticos adversos no câncer colorretal e estão incorporados como N1c no sistema de estadiamento TNM. No entanto, sua relevância no câncer gástrico (CG) permanece pouco clara.

**Objetivo:**

Este estudo teve como objetivo investigar a associação entre DTs, características patológicas e desfechos de sobrevida no CG.

**Métodos:**

Realizamos um estudo transversal com pacientes com diagnóstico histopatológico confirmado de CG tratados em dois hospitais terciários em Tabriz, Irã, entre janeiro de 2020 e dezembro de 2024. Foram coletados dados clínicos e patológicos, incluindo características tumorais e estado de sobrevida. Os DTs e suas características também foram avaliados. As associações entre a presença de DTs e as características clinicopatológicas foram analisadas por modelos de regressão logística, enquanto a sobrevida global foi avaliada por análise de Kaplan-Meier e regressão de riscos proporcionais de Cox.

**Results:**

O estudo incluiu 256 pacientes (idade média 60,7±12,3 anos; 61,3% do sexo masculino). DTs estavam presentes em 111 pacientes (43,4%). Na análise multivariável, a categoria pT (OR=7,44; IC95%: 2,22-24,89), a categoria pN (OR=4,63; IC95%: 1,97-10,86) e a invasão perineural (OR=5,65; IC95%: 2,40-13,29) foram significativamente associadas aos DTs. A análise de Kaplan-Meier mostrou taxas de sobrevida global em 1, 3 e 5 anos de 93,7%, 75,7% e 64,9%, respectivamente. Pacientes sem DTs apresentaram sobrevida significativamente melhor em comparação com aqueles com DTs (log-rank *P*<0,001). Na regressão de Cox multivariável, subtipo histológico (HR=2,07; IC95%: 1,21-3,52), categoria pT (HR=4,68; IC95%: 1,37-16,00), categoria pN (HR=2,79; IC95%: 1,21-6,46) e presença de DTs (HR=2,00; IC95%: 1,06-3,75) foram preditores independentes de pior sobrevida global.

**Conclusion:**

Os DTs foram observados em uma proporção considerável de casos de CG e estiveram associados a características patológicas mais agressivas e pior sobrevida. Eles podem melhorar a estratificação prognóstica, mas necessitam de validação adicional em estudos multicêntricos maiores.

## INTRODUCTION

Gastric cancer (GC) remains a major global health burden and is among the leading causes of cancer-related mortality worldwide^1^. Despite advances in diagnosis and treatment, long-term outcomes remain poor, largely due to late-stage presentation and diverse metastatic pathways^2^. Prognostic stratification and therapeutic decision-making in GC still rely mainly on clinicopathological features and molecular markers^3^. However, the substantial heterogeneity of the disease limits their predictive accuracy, highlighting the continued need for complementary prognostic indicators^5^.

One proposed marker of aggressive disease is the presence of tumor deposits (TD)^5^ ^,^ ^6^, defined as discrete nodules of carcinoma in the perigastric fat that lack identifiable lymph node, vascular, or neural tissue^7^ ^,^ ^8^. Although TDs were first described in colorectal cancer^9^, they have since been reported in several other malignancies, including GC^10^ ^,^ ^11^. In colorectal cancer TDs are classified as N1c in TNM staging^12^, yet in GC they are not formally incorporated in the AJCC/UICC classification^6^ ^,^ ^8^. Existing evidence suggests that TDs occur in roughly 10-30% of gastric resections and are associated with advanced disease^5^ ^,^ ^13^ ^,^ ^14^. For example, Lee et al. found TDs in 23.9% of 653 GC patients, with TDs correlating with synchronous metastasis and independently predicting worse overall survival^14^. A recent meta-analysis similarly showed that the occurrence of TDs (mean ~21%) was associated with higher T and N categories and poorer survival (multivariable HR ~1.65 for OS)^13^.

Despite accumulating data, the clinicopathological factors that are associated with TDs and their precise impact on patient prognosis remain under debate^7^ ^,^ ^10^ ^,^ ^15^. Some studies have identified advanced tumor stage and nodal involvement as correlates^7^ ^,^ ^13^, but reports vary, and not all potential pathological features have been examined^8^ ^,^ ^10^ ^,^ ^14^ ^,^ ^16^. This study aims to investigate the association between TDs and both pathological characteristics and survival outcomes in patients with GC.

## METHODS

This retrospective cohort study included all patients with a pathological diagnosis of GC who were treated at Sina and Imam Reza Hospitals, Tabriz, between January 2020 and December 2024. During this period, 280 patients were initially identified. After applying the exclusion criteria, namely incomplete medical or pathological records or unavailable tissue samples, 24 patients were excluded, resulting in a final study cohort of 256 patients.

A post-hoc power calculation based on the observed proportion of TDs and the effect sizes from multivariable logistic regression indicated that the study had >80% power to detect the observed odds ratios at a two-sided alpha of 0.05.

Data were extracted from participants’ medical and pathological records. The collected variables comprised: 1) demographic data, including sex and age at diagnosis; 2) clinicopathological features of the primary tumor, including tumor location, histological subtype, histological grade, pathological tumor stage (TNM), perineural invasion, lymphovascular invasion, lymph node metastasis, type of surgery, type of lymphadenectomy, number of examined lymph nodes, surgical margin status, neoadjuvant therapy, and tumor size; 3) TD characteristics, including presence, pattern, margin, size, and number; and 4) clinical outcomes, including overall survival status and survival time. Survival time was defined as the interval (in months) from the date of surgery to death or last follow-up.

All pathology slides were re-evaluated independently by two experienced pathologists. In cases of disagreement, the final diagnosis was established after consultation with a third pathologist. Pathological assessment was performed according to the 8th edition of the UICC/AJCC TNM classification system. TDs were defined as discrete nodules of tumor cells located in the peritumoral or perigastric adipose tissue, discontinuous from the primary tumor and lacking identifiable lymph node, vascular, or nerve structures. Lesions located within vascular or lymphatic structures were classified as lymphovascular invasion, and those adjacent to nerve structures as perineural invasion^14^ ^-^ ^16^.

Participants were classified into two groups based on the presence of TDs. The associations of TDs with primary tumor clinicopathological features were evaluated using univariable and multivariable logistic regression models. Overall survival was analyzed using the Kaplan-Meier method, and survival curves were compared between groups using the log-rank test. Estimated overall survival rates at 1, 3, and 5 years were calculated. Univariable and multivariable Cox proportional hazards regression analyses were performed to identify factors associated with overall survival, and hazard ratios (HRs) with 95% confidence intervals (CIs) were reported. Statistical analyses were conducted using SPSS software (version 25.0), and a *P*-value <0.05 was considered statistically significant.

This study was conducted in accordance with the Declaration of Helsinki and relevant national regulations. The study protocol was reviewed and approved by the Ethics Committee of Tabriz University of Medical Sciences (approval number: 1402.221; approval date: 12 June 2023). The requirement for written informed consent was waived by the ethics committee because of the retrospective nature of the study, and all data were anonymised to protect participant confidentiality.

This study was reported in accordance with the RECORD statement for observational studies^17^.

## RESULTS

A total of 256 patients were included in the analysis. The mean age was 60.7 years (SD, 12.3), and 61.3% were male. TDs were present in 43.4% of cases. The remaining baseline clinicopathological characteristics are summarized in Table 1.

**TABLE 1:** Clinicopathological characteristics of the patients included in the study.

Variable		n (%)
Sex	Female	99 (38.7)
	Male	157 (61.3)
Age	<60 years	109 (42.6)
	≥60 years	147 (57.4)
Tumor location	Distal	79 (30.9)
	Middle	112 (43.8)
	Upper	65 (25.4)
Histological subtype	Intestinal	147 (57.4)
	Diffuse	85 (33.2)
	Mixed	24 (9.4)
Histological grade	Differentiated	226 (88.3)
	Undifferentiated	30 (11.7)
pT	pTis	7 (2.7)
	pT1a	9 (3.5)
	pT1b	20 (7.8)
	pT2	44 (17.2)
	pT3	80 (31.3)
	pT4a	91 (35.5)
	pT4b	5 (2.0)
pN	pN0	96 (37.5)
	pN1	25 (9.8)
	pN2	39 (15.2)
	pN3a	79 (30.9)
	pN3b	17 (6.6)
pM	pMx	254 (99.2)
	pM1	2 (0.8)
pTNM	Stage 0	7 (2.7)
	Stage IA	25 (9.8)
	Stage IB	28 (10.9)
	Stage IIA	32 (12.5)
	Stage IIB	26 (10.2)
	Stage IIIA	23 (9.0)
	Stage IIIB	54 (21.1)
	Stage IIIC	59 (23.0)
	Stage IV	2 (0.8)
Perineural invasion	Absent	97 (37.9)
	Present	159 (62.1)
Lymphovascular invasion	Absent	81 (31.6)
	Present	175 (68.4)
Lymph node metastasis	Absent	95 (37.1)
	Present	161 (62.9)
Type of surgery	Subtotal	91 (35.5)
	Total	165 (64.5)
Type of lymphadenectomy	D1	50 (19.5)
	D2	206 (80.5)
No. of examined lymph nodes	<16	45 (17.6)
	≥16	211 (82.4)
Surgical margin	R0	226 (88.3)
	R1/R2	30 (11.7)
Neoadjuvant therapy	No	174 (68.0)
	Yes	82 (32.0)
Tumor size	<60 mm	153 (59.8)
	≥60 mm	103 (40.2)
Tumor deposit (TD)	Absent	145 (56.6)
	Present	111 (43.4)
TD pattern	Nodular	55 (49.5)^*^
	Perilymphovascular	52 (46.8)^*^
	Perineural	4 (3.6)^*^
TD margin	Regular	63 (56.8)^*^
	Irregular	48 (43.2)^*^
TD size	0.5 - 2 mm	61 (55.0)^*^
	2 - 5 mm	50 (45.0)^*^
TD number	1	16 (14.4)^*^
	2 or more	95 (85.6)^*^
Survival status	Alive	144 (64.9)^†^
	Deceased	78 (35.1)^†^

Percentages are calculated based on all patients (n=256), unless otherwise indicated. ^*^Percentages for tumor deposit characteristics are calculated among patients with tumor deposits (n=111). ^†^Percentages for patient survival status are calculated among patients with available survival data (n=222).

To evaluate factors associated with the presence of TDs, univariable and multivariable analyses were conducted. In univariable analysis, the presence of TDs was significantly associated with tumor location, histological subtype, pT category, pN category, perineural invasion, lymphovascular invasion, type of surgery, and tumor size. In multivariable analysis, the independent predictors of TDs were pT category (OR=7.44, 95%CI: 2.22-24.89, *P*=0.001), pN category (OR=4.63, 95%CI: 1.97-10.86, *P*<0.001), and perineural invasion (OR=5.65, 95%CI: 2.40-13.29, *P*<0.001) (Table 2).

**TABLE 2:** Univariable and multivariable logistic regression analyses of factors associated with the presence of tumor deposits.

Variable		Univariable analysis		Multivariable analysis	
		Odds ratio (95%CI)	*P*	Odds ratio (95%CI)	*P*
Sex	Female	1 (ref)			
	Male	1.218 (0.731 - 2.028)	0.449		
Age	<60 years	1 (ref)			
	≥60 years	0.735 (0.446 - 1.212)	0.227		
Tumor location	Distal	1 (ref)	0.031	1 (ref)	0.791
	Middle	2.239 (1.226 - 4.086)	0.009	1.331 (0.503 - 3.523)	0.565
	Upper	1.740 (0.880 - 3.439)	0.111	1.073 (0.361 - 3.187)	0.899
Histological subtype	Intestinal	1 (ref)		1 (ref)	
	Non-intestinal	1.767 (1.069 - 2.922)	0.026	0.898 (0.433 - 1.861)	0.772
Histological grade	Differentiated	1 (ref)			
	Undifferentiated	1.164 (0.542 - 2.498)	0.697		
pT	pTis, pT1, pT2	1 (ref)		1 (ref)	
	pT3, pT4	29.464 (10.311 - 84.197)	*P*<0.001	7.440 (2.224 - 24.887)	**0.001**
pN	pN0	1 (ref)		1 (ref)	
	pN1, pN2, pN3	11.361 (5.733 - 22.511)	*P*<0.001	4.628 (1.972 - 10.862)	***P*<0.001**
Perineural invasion	Absent	1 (ref)		1 (ref)	
	Present	13.251 (6.546 - 26.826)	*P*<0.001	5.649 (2.402 - 13.286)	***P*<0.001**
Lymphovascular invasion	Absent	1 (ref)		1 (ref)	
	Present	4.824 (2.588 - 8.991)	*P*<0.001	1.832 (0.776 - 4.325)	0.167
Type of surgery	Subtotal	1 (ref)		1 (ref)	
	Total	2.458 (1.427 - 4.233)	0.001	0.854 (0.326 - 2.234)	0.748
Adjuvant therapy	No	1 (ref)			
	Yes	0.892 (0.524 - 1.518)	0.674		
Tumor size	<60 mm	1 (ref)		1 (ref)	
	≥60 mm	2.432 (1.457 - 4.059)	0.001	1.337 (0.650 - 2.750)	0.430

**FIGURE 1:**
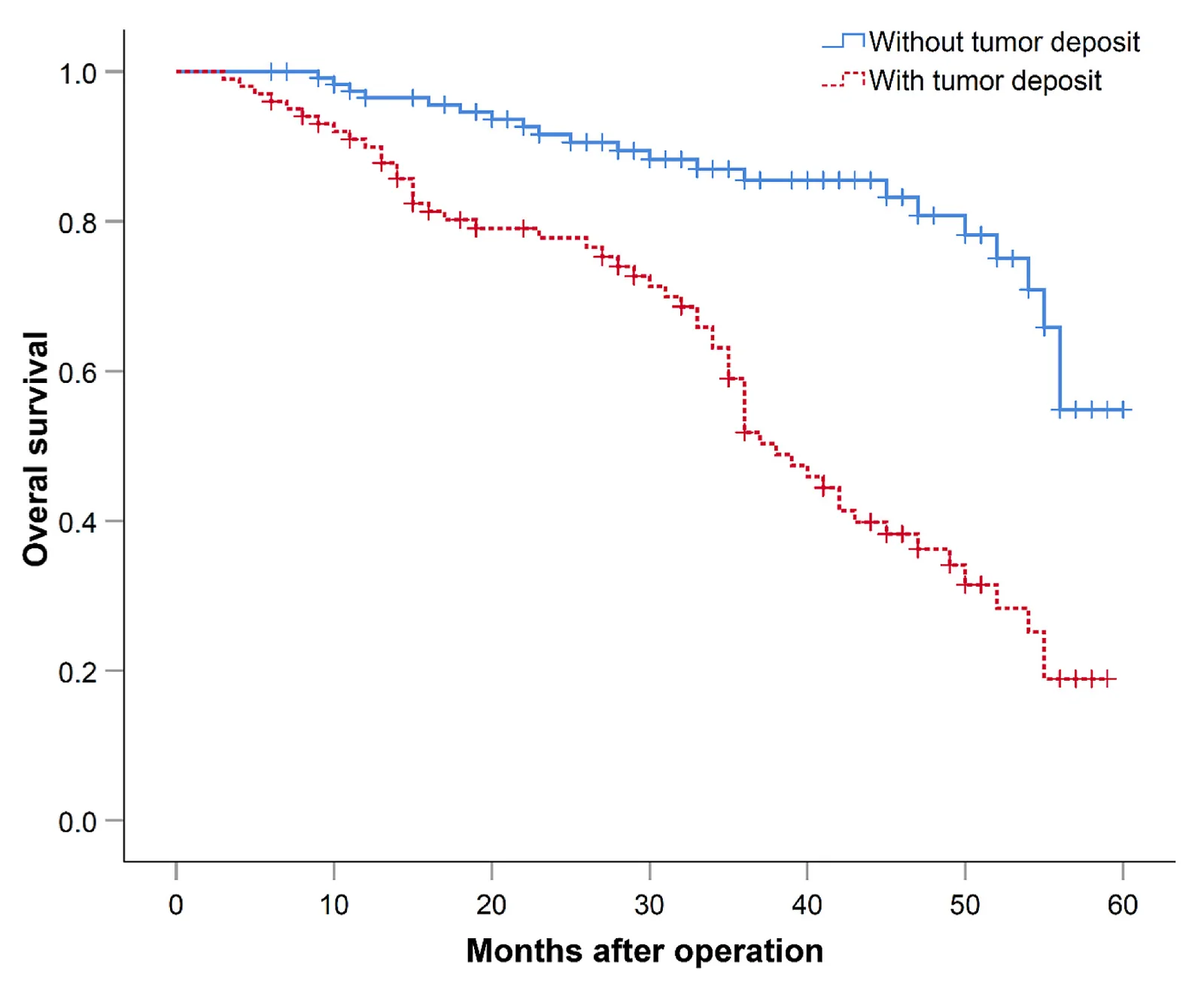
Kaplan-Meier survival curves comparing overall survival between patients with and without tumor deposits.

Kaplan-Meier survival analysis demonstrated that the estimated overall survival rates for the entire cohort at 1, 3, and 5 years were 93.7%, 75.7%, and 64.9%, respectively. When stratified by TD status, patients without TDs had significantly longer survival compared to those with TDs. The mean survival time was 52.4 months (95%CI: 49.6-55.2) in the TD-negative group and 38.0 months (95%CI: 34.4-41.6) in the TD-positive group. The difference in survival distributions between the two groups was statistically significant, as demonstrated by the log-rank test (χ^2^=32.1, *P*<0.001), as well as Breslow and Tarone-Ware tests (both *P*<0.001), indicating a significantly poorer survival in patients with TDs (Figure 1).

In univariable Cox regression analyses, overall survival was significantly associated with histological subtype, histological grade, pT and pN categories, perineural invasion, lymphovascular invasion, type of surgery, tumor size, and the presence of TDs. In the multivariable Cox proportional hazards model, histological subtype (HR=2.07, 95%CI: 1.21-3.52, *P*=0.007), pT category (HR=4.68, 95%CI: 1.37-16.00, *P*=0.014), pN category (HR=2.79, 95%CI: 1.21-6.46, *P*=0.016), and the presence of TD (HR=2.00, 95%CI: 1.06-3.75, *P*=0.032) remained independent predictors of poorer overall survival (Table 3).

**TABLE 3:** Univariable and multivariable Cox proportional hazards regression analyses of factors associated with overall survival.

Variable		Univariable analysis		Multivariable analysis	
		Hazard Ratio (95%CI)	*P*	Hazard Ratio (95%CI)	*P*
**Sex**	Female	1 (ref)			
	Male	1.050 (0.665 - 1.658)	0.834		
**Age**	<60 years	1 (ref)			
	≥60 years	0.834 (0.533 - 1.307)	0.429		
**Tumor location**	Distal	1 (ref)	0.437		
	Middle	1.409 (0.803 - 2.471)	0.232		
	Upper	1.423 (0.762 - 2.655)	0.268		
**Histological subtype**	Intestinal	1 (ref)		1 (ref)	
	Non-intestinal	3.215 (2.018 - 5.120)	*P*<0.001	2.067 (1.214 - 3.517)	0.007
**Histological grade**	Differentiated	1 (ref)		1 (ref)	
	Undifferentiated	2.053 (1.144 - 3.686)	0.016	1.338 (0.668 - 2.677)	0.411
**pT**	pTis, pT1, pT2	1 (ref)		1 (ref)	
	pT3, pT4	12.340 (4.494 - 33.889)	*P*<0.001	4.678 (1.368 - 16.003)	0.014
**pN**	pN0	1 (ref)		1 (ref)	
	pN1, pN2, pN3	7.994 (3.840 - 16.641)	*P*<0.001	2.792 (1.206 - 6.461)	0.016
**Perineural invasion**	Absent	1 (ref)		1 (ref)	
	Present	3.437 (1.893 - 6.243)	*P*<0.001	0.774 (0.324 - 1.852)	0.565
**Lymphovascular invasion**	Absent	1 (ref)		1 (ref)	
	Present	2.588 (1.426 - 4.698)	0.002	1.184 (0.606 - 2.315)	0.620
**Type of surgery**	Subtotal	1 (ref)		1 (ref)	
	Total	1.972 (1.165 - 3.341)	0.012	1.512 (0.860 - 2.659)	0.151
**Adjuvant therapy**	No	1 (ref)			
	Yes	0.750 (0.442 - 1.271)	0.285		
**Tumor size**	<60 mm	1 (ref)		1 (ref)	
	≥60 mm	3.922 (2.401 - 6.406)	*P*<0.001	1.567 (0.882 - 2.783)	0.125
**Tumor deposit**	Absent	1 (ref)		1 (ref)	
	Present	3.754 (2.289 - 6.157)	*P*<0.001	1.997 (1.063 - 3.751)	0.032

## DISCUSSION

In this study, TDs were frequently observed in patients with GC and were significantly associated with aggressive pathological features, including deeper invasion, higher nodal stage, and perineural invasion. Furthermore, TDs, along with tumor depth, nodal involvement, and histological subtype, were significant predictors of overall survival. This finding highlights their potential prognostic significance.

The frequency of TDs in our study was 43.4%, which is higher than previously reported rates of 10.5-27.5% in China^6^ ^,^ ^8^ ^,^ ^15^, 24-26.2% in Turkey^4^ ^,^ ^18^, and 23.9% in South Korea^14^. This discrepancy may be influenced by various factors, including demographic characteristics, methodological differences, and pathological assessment techniques. Differences in histopathological features, such as a higher proportion of advanced-stage disease or more aggressive histological subtypes, may play a particularly important role.

In analyzing the clinicopathological correlates of TDs, the univariable results showed that TD presence was significantly associated with proximal tumor location, non-intestinal histology, greater tumor depth (pT stage), higher nodal stage (pN stage), perineural invasion, lymphovascular invasion, total gastrectomy, and larger tumor size. In multivariable logistic regression, only deeper invasion (pT stage), higher nodal metastasis rate (pN stage), and perineural invasion remained independent predictors of TD. This suggests that TD-positive tumors tend to be more advanced and invasive, even after adjustment for other covariates.

The higher frequency of TDs in proximal compared with distal GCs is consistent with prior studies, which reported that tumors in the upper and middle third of the stomach often exhibit more aggressive behavior, including deeper invasion and higher rates of lymphovascular permeation^6^ ^,^ ^10^. Anatomically, proximal gastric tumors are located adjacent to extensive lymphovascular networks^19^, which may predispose them to discontinuous spread and increase the likelihood of TD formation.

With respect to histological subtype, our finding that TDs were more frequent in non-intestinal tumors parallels the report of Lee et al.^14^ although Ersen et al.^4^ observed the opposite trend. Our result is consistent with the view that diffuse-type GCs often display infiltrative growth and a greater tendency toward peritoneal dissemination and microscopic spread along tissue planes^20^.

The significant association of TDs with features of advanced disease such as deep invasion, nodal involvement, and large primary tumor size is in line with prior evidence. Torres et al. reported that tumor size ≥5 cm, serosal invasion (pT4), and extensive nodal metastasis (pN3b) were independent risk factors for TDs in GC^7^. Similarly, a propensity-matched study showed that TDs were significantly more common in tumors with advanced T and N stage^10^. A meta-analysis showed that TDs are significantly associated with higher T category and nodal positivity^13^. Together, these findings support the interpretation that TDs may arise as a manifestation of aggressive, deeply invasive cancers.

Our observation that TDs were more frequent in cases requiring total rather than subtotal gastrectomy is also in agreement with the studies by Gu et al.^8^ and Liang et al.^6^. This may partly reflect more extensive disease necessitating wider surgical margins. Alternatively, it may indicate that cases with TD generally present with a higher tumor burden, which could necessitate more extensive resections.

Of note, in our multivariable analysis, perineural invasion emerged as an independent correlate of TD. Perineural invasion is a recognized marker of tumor aggressiveness and a conduit for local spread^21^. Its independent association with TD raises the possibility that tumors infiltrating along nerve sheaths may also disseminate malignant cells into adjacent adipose tissue, further supporting the hypothesis that TD represent an extranodal metastatic pathway^11^.

Notably, not all factors remained significant in multivariable analysis. For example, lymphovascular invasion, diffuse histology, and tumor size were more common in TD-positive cases in univariable testing, but did not independently predict TD in multivariable analysis. This suggests that tumor depth, nodal burden, and perineural invasion may play a greater role in determining TD presence.

With respect to overall survival, histological subtype, pT category, pN category, and TD presence emerged as significant prognostic factors in our multivariable Cox analysis.

The adverse prognostic impact of diffuse histology, deeper tumor invasion, and higher nodal stage is well established in GC and has been consistently reported in previous studies^22^ ^,^ ^23^. These variables are widely recognized as important predictors of disease progression and patient outcomes, and therefore they provide an appropriate context for focusing on the prognostic implications of TD.

Our finding that TD presence was associated with worse survival is consistent with the growing body of evidence suggesting that TDs represent an adverse prognostic feature in GC. Lee et al.^14^ reported significantly reduced survival in TD-positive patients. A systematic review likewise found that TD presence was associated with lower overall survival^13^ Song et al.^10^ demonstrated a 5-year survival rate of 31.0% for TD-positive versus 60.9% for TD-negative patients and confirmed TD as an independent predictor of mortality. Similarly, Zhou et al.^15^ showed that TD presence independently shortened both disease-free and overall survival, and Tan et al.^16^ found markedly reduced survival in TD-positive patients, to a degree comparable with pN3 disease. Together, these findings reinforce the prognostic significance of TD across diverse cohorts.

From a clinical perspective, the presence of TDs may have important implications for treatment decision-making. Given their association with advanced disease and poorer survival, TD-positive patients may represent a higher-risk subgroup who could benefit from more aggressive adjuvant chemotherapy strategies. Although current treatment guidelines for gastric cancer are primarily based on TNM staging, emerging evidence suggests that additional pathological features, including TDs, may refine risk stratification and help guide postoperative management^8^ ^,^ ^24^. However, prospective studies are needed to determine whether TD status should be formally incorporated into therapeutic decision-making algorithms.

In addition to conventional clinicopathological factors, emerging evidence suggests that molecular heterogeneity may also influence the prognosis of gastric cancer. Molecular subtypes such as Epstein-Barr virus (EBV)-positive and microsatellite instability-high (MSI-H) tumors represent distinct biological entities with unique tumor microenvironments and immune profiles. Some studies have reported that these subtypes may be associated with better prognosis or differential treatment response, although findings remain inconsistent across cohorts^25^ ^-^ ^27^. It is therefore conceivable that the prognostic impact of TDs may be modulated by underlying molecular characteristics, a hypothesis that warrants further investigation.

A possible explanation for these observations is that TDs may represent a distinct mode of tumor spread not fully captured by conventional nodal staging. Their presence may reflect lymphovascular or perineural invasion, or discontinuous tumor dissemination within the perigastric tissues, potentially occurring regardless of overt nodal metastases^11^ ^,^ ^28^ ^-^ ^30^. This may complicate locoregional control and worsen prognosis. This concept is well established in colorectal cancer, where TDs have been incorporated into the AJCC staging system because of their strong prognostic value^28^. Although this aspect has been less studied in GC, recent reports similarly suggest that TDs may function as an independent adverse prognostic factor. Accordingly, consideration of TDs in the TNM staging system for GC has gained increasing attention^6^ ^,^ ^8^ ^,^ ^10^ ^,^ ^15^.

This study has several limitations. As a retrospective analysis, it is subject to inherent biases such as selection and information bias, and it cannot establish causality. The assessment of TDs was performed through pathology review by two pathologists, with a third consulted in cases of disagreement. Although this approach helps reduce interobserver variability, some degree of subjectivity in identifying TDs cannot be excluded. We did not have survival data for all patients, which could introduce bias. We also lacked complete information on patients’ comorbidities and risk factors, which could have influenced outcomes. Our patients were drawn from two hospitals in Iran, which may limit generalizability. Finally, our sample size was relatively small, which may reduce the statistical power and limit the robustness of our findings.

## CONCLUSION

TDs were observed in a considerable proportion of GC cases and were associated with more aggressive pathological features and poorer survival. These findings suggest that incorporating TDs into routine pathological assessment may improve prognostic stratification, but their formal integration into staging systems requires further validation. Future prospective, multicenter studies with larger cohorts and comprehensive clinical data are needed to confirm and extend these findings.

## Data Availability

Data available upon request.

## References

[1] Bray F, Laversanne M, Sung H, Ferlay J, Siegel RL, Soerjomataram I (2024). Global cancer statistics 2022: GLOBOCAN estimates of incidence and mortality worldwide for 36 cancers in 185 countries. CA: a cancer journal for clinicians.

[2] Sundar R, Nakayama I, Markar SR, Shitara K, van Laarhoven HW, Janjigian YY (2025). Gastric cancer. The Lancet.

[3] Guan W-L, He Y, Xu R-H (2023). Gastric cancer treatment: recent progress and future perspectives. Journal of hematology oncology.

[4] Ersen A, Unlu MS, Akman T, Sagol O, Oztop I, Atila K (2014). Tumor deposits in gastric carcinomas. Pathology-Research Practice.

[5] Hayashi M, Abe M, Fujita T, Matsushita H (2024). Prognostic effect of categorized tumor deposits in gastric cancer: A single-center retrospective study. Surgery.

[6] Liang Y, Wu L, Liu L, Ding X, Wang X, Liu H (2019). Impact of extranodal tumor deposits on prognosis and N stage in gastric cancer. Surgery.

[7] Torres OP, Cucho SP, Rojas LT, Luque-Vasquez C, Chavez I, Meza EP (2021). Clinicopathological factors associated with the presence of tumor deposits in resected gastric cancer patients. Heliyon.

[8] Gu L, Chen P, Su H, Li X, Zhu H, Wang X (2020). Clinical significance of tumor deposits in gastric cancer: a retrospective and propensity score-matched study at two institutions. Journal of Gastrointestinal Surgery.

[9] Ueno H, Nagtegaal ID, Quirke P, Sugihara K, Ajioka Y (2023). Tumor deposits in colorectal cancer: Refining their definition in the TNM system. Annals of Gastroenterological Surgery.

[10] Song X, Liu K, Liao X, Zhu Y, Peng B, Zhang W (2023). Clinical significance of tumor deposits in gastric cancer after radical gastrectomy: a propensity score matching study. World Journal of Surgical Oncology.

[11] Sarioglu S (2018). Tumor deposits; mechanisms, morphology, and differential diagnosis. Tumor Deposits.

[12] Delattre J-F, Erdogan ASO, Cohen R, Shi Q, Emile J-F, Taieb J (2022). A comprehensive overview of tumour deposits in colorectal cancer: Towards a next TNM classification. Cancer treatment reviews.

[13] Graham Martínez C, Knijn N, Verheij M, Nagtegaal ID, van der Post RS (2019). Tumour deposits are a significant prognostic factor in gastric cancer-a systematic review and meta‐analysis. Histopathology.

[14] Lee HS, Lee HE, Yang H-K, Kim WH (2013). Perigastric tumor deposits in primary gastric cancer: implications for patient prognosis and staging. Annals of surgical oncology.

[15] Zhou M, Yang W, Zou W, Yang J, Zhou C, Zhang Z (2022). Prognostic significance of tumor deposits in radically resected gastric cancer: a retrospective study of a cohort of 1915 Chinese individuals. World Journal of Surgical Oncology.

[16] Tan J, Yang B, Xu Z, Zhou S, Chen Z, Huang J (2019). Tumor deposit indicates worse prognosis than metastatic lymph node in gastric cancer: a propensity score matching study. Annals of Translational Medicine.

[17] Benchimol EI, Smeeth L, Guttmann A, Harron K, Moher D, Petersen I (2015). The REporting of studies Conducted using Observational Routinely-collected health Data (RECORD) statement. PLoS medicine.

[18] Yildiz B, Etiz D, Dal P, Junushova B, Pasaoglu O, Yilmaz E (2016). Tumor deposits: prognostic significance in gastric cancer patients. J buon.

[19] Kinami S, Nakamura N, Miyashita T, Kitakata H, Fushida S, Fujimura T (2021). nPTD classification: an updated classification of gastric cancer location for function preserving gastrectomy based on physiological lymphatic flow. BMC cancer.

[20] Del Arco CD, Muñoz LE, Medina LO, Roldán EM, Nieto MAC, de Las Heras SGG (2022). Clinicopathological differences, risk factors and prognostic scores for western patients with intestinal and diffuse-type gastric cancer. World Journal of Gastrointestinal Oncology.

[21] Liu Q, Ma Z, Cao Q, Zhao H, Guo Y, Liu T (2022). Perineural invasion-associated biomarkers for tumor development. Biomedicine Pharmacotherapy.

[22] Mantziari S, St Amour P, Abboretti F, Teixeira-Farinha H, Gaspar Figueiredo S, Gronnier C (2023). A comprehensive review of prognostic factors in patients with gastric adenocarcinoma. Cancers.

[23] Kulig P, Pach R, Majewska O, Kulig J (2022). Clinicopathological prognostic factors determining outcomes of treatment in gastric cancer surgery. in vivo.

[24] Lordick F, Carneiro F, Cascinu S, Fleitas T, Haustermans K, Piessen G (2022). Gastric cancer: ESMO Clinical Practice Guideline for diagnosis, treatment and follow-up. Annals of Oncology.

[25] Network CGAR (2014). Comprehensive molecular characterization of gastric adenocarcinoma. Nature.

[26] Smyth EC, Nilsson M, Grabsch HI, van Grieken NCT, Lordick F (2020). Gastric cancer. The Lancet.

[27] Polom K, Marano L, Marrelli D, De Luca R, Roviello G, Savelli V (2018). Meta-analysis of microsatellite instability in relation to clinicopathological characteristics and overall survival in gastric cancer. Br J Surg.

[28] Nagtegaal ID, Knijn N, Hugen N, Marshall HC, Sugihara K, Tot T (2017). Tumor deposits in colorectal cancer: improving the value of modern staging-a systematic review and meta-analysis. Journal of Clinical Oncology.

[29] Xu R, Zhang Y, Zhao J, Chen K, Wang Z (2023). Prognostic value of tumor deposits in lymph node-negative gastric cancer: a propensity score matching study. European Journal of Surgical Oncology.

[30] Anup S, Lu J, Zheng C-H, Li P, Xie J-W, Wang J-B (2017). Prognostic significance of perigastric tumor deposits in patients with primary gastric cancer. BMC surgery.

